# Relative expected value of drugs versus competing rewards underpins vulnerability to and recovery from addiction

**DOI:** 10.1016/j.bbr.2020.112815

**Published:** 2020-09-15

**Authors:** Lee Hogarth, Matt Field

**Affiliations:** a Lee Hogarth, School of Psychology, University of Exeter, Washington Singer Building, Perry Road, Exeter EX4 4QG, UK; b Department of Psychology, University of Sheffield

**Keywords:** Concurent drug choice tasks, Relative value, Economic decision theory, Addiction

## Abstract

Behavioural economic theories of addiction contend that greater expected value of drug relative to alternative non-drug rewards is the core mechanism underpinning vulnerability to and recovery from addiction. To evaluate this claim, we exhaustively review studies with human drug users that have measured concurrent choice between drugs vs. alternative rewards, and explored individual differences. These studies show that drug choice can be modulated by drug cues, drug devaluation, imposition of costs/punishment and negative mood induction. Regarding individual differences, dependence severity was reliably associated with overall drug preference, and self-reported drug use to cope with negative affect was reliably associated with greater sensitivity to mood induced increases in drug choice. By contrast, there were no reliable individual differences in sensitivity to the effect of drug cues, drug devaluation or punishment on drug choice. These findings provide insight into the mechanisms that underpin vulnerability to dependence: vulnerability is conferred by greater relative value ascribed to drugs, and relative drug value is further augmented by negative affective states in those who report drug use coping motives. However, dependence does not appear to be characterised by abnormal cue-reactivity, habit learning or compulsion. We then briefly review emerging literature which demonstrates that therapeutic interventions and recovery from addiction might be attributed to changes in the expected relative value of drug versus alternative rewards. Finally, we outline a speculative computational account of the distortions in decision-making that precede action selection in addiction, and we explain how this account provides a blueprint for future research on the determinants of drug choice, and mechanisms of treatment and recovery from addiction. We conclude that a unified economic decision-making account of addiction has great promise in reconciling diverse addiction theories, and neuropsychological evaluation of the underlying decision mechanisms is a fruitful area for future research and treatment.

## 1 Reconciling relative value and dual-process theories of addiction

Behavioural economic theories of addiction contend that the relative value of drug compared to non-drug rewards is a core mechanism underpinning drug dependence [[1], [2], [3], [4], [5], [6], [7], [8], [9], [10], [11]]. Although most theories accept that the drug’s relative reward value plays a role, particularly in recreational or initial use, many theories go on to postulate a secondary process that overrides or summates with reward learning to create the ‘addicted’ state. One of the earliest dual-process models was negative reinforcement theory, which claims that growth in the withdrawal syndrome, psychiatric symptoms or negative affect further motivates drug use in order to alleviate these states [12]. Cue-reactivity theories inspired by associative learning contend that through Pavlovian conditioning, drug associated cues become capable of eliciting drug-seeking automatically [13] or via an expectancy of drug availability [14]. Relatedly, habit theories of addiction argue that drug reinforcement stamps in strong S-R associations such that drug related stimuli (S) elicit drug-seeking responses (R) *autonomously*, i.e. without forethought of the consequences [15,16]. Compulsion theories go further and claim that S-R associations become maladaptive (fixed) such that they can no longer be modified by direct experience of punishment or harmful consequences (unlike habitual S-R/reinforcement learning which can be modified by experience of changed outcome value) [17]. Finally, neurocognitive accounts argue that chronic drug use produces structural and functional brain changes [18] and thus cognitive deficits [19], including impaired inhibitory control [20] and impaired foresight of future rewards and costs [21], rendering drug-seeking less susceptible to change by decision making.

Unified economic decision making accounts of addiction have attempted to reconcile these competing theories by suggesting that all of the secondary processes might promote drug-seeking via their impact on the expected relative value of the drug compared to competing non-drug alternative rewards, that is, by biasing preferential choice towards drugs [5,6,10,22,23]. On this view, addiction is driven by a single process – the relative expected value ascribed to the drug versus to non-drug rewards – but this process can be influenced by a multitude of factors. Although this unified decision framework has an attractive breadth of explanatory power, because predictions derived from all theoretical models can be incorporated into one, it remains to be seen whether the unified account can be justified with evidence.

The current article evaluates the unified economic decision model of addiction in three sections, before outlining future directions. First, we consider whether the relative value of drugs vs. alternatives can explain vulnerability to dependence. An exhaustive review is provided of human concurrent choice studies which have tested whether choice between drugs vs. alternative rewards can be modified by experimental manipulations (cues, devaluation, costs and negative affect) and whether these effects are associated with self-reported dependence severity and associated risk factors (psychiatric symptoms and drug use to cope with negative affect). This work indicates that dependence severity is reliably associated with greater relative drug value (preferential drug choice), and drug use to cope with negative affect is reliably associated with greater sensitivity to negative affect induced increases in relative drug value, suggesting at least two risk pathways converging on relative drug value. In the remainder of the paper we offer a brief overview of emerging research findings which demonstrate that recovery from addiction might be explained by changes in the relative value of the drug versus alternative rewards. Finally, we outline a speculative computational account that describes the internal processes that underlie valuation of drug and alternative rewards as a precursor to behavioural choice. Overall, we believe that the unified economic decision-making account has great promise in explaining both vulnerability to and recovery from addiction, and that neuropsychological evaluation of the underlying decision mechanisms would be a fruitful area for future research.

## 2 Relative value of drugs versus alternative rewards underpins vulnerability to dependence

Evidence that addiction is driven by expected relative drug value comes from the finding that dependence severity in humans, assessed by questionnaires or diagnostic criteria, is reliably associated with greater economic demand for drugs. In economic demand tasks, participants report their hypothetical consumption of a drug across a range of prices, indexing the relative value of the drug compared to money (money is exchangeable for most rewards). This index of drug value has been shown to correlate with dependence severity in 41 studies on alcohol demand, 34 studies on nicotine demand and 10 studies on other substance demand, in both clinical and non-clinical samples [3,24]. Although these associations are largely cross sectional, they nevertheless provide compelling evidence supporting the claim that human drug dependence severity is underpinned by greater relative value ascribed to the drug compared to alternative rewards.

Human concurrent choice tasks have also indexed the value ascribed to drugs relative to money and other rewards (e.g. food). In the concurrent choice task, participants make a choice between the drug and another reward across a series of trials, and the proportion of drug choices indexes the relative value of the drug versus the alternative reward [7,25]. Choices may earn points for their respective rewards in a token economy, or pictures of the rewards (reviewed in Table 1, Table 2, Table 3). Actual consumption of chosen rewards has sometimes been arranged, but individual differences analysis has not been undertaken in such studies, perhaps because of the complexity and low throughput of the method [[26], [27], [28], [29], [30], [31], [32]] but see [33]. Finally, animal concurrent choice procedures have also been used to study the neuropsychological basis of relative drug value [34].

**Table 1:** List of studies which have used concurrent choice tasks to measure the preference between a drug versus an alternative reward in a drug user sample, and tested whether dependence severity is associated with drug choice in a baseline condition (column 5), and/or with the change in drug choice produced by drug cues (column 6), drug devaluation (column 7), the imposition of costs (column 8), or negative mood induction (column 9). Column 1 reports the paper, where E1/E2 = experiment number. Column 2 reports the drug user group tested. Column 3 reports the sample size. Column 4 reports the type of choice task: TE = token economy; PC = pictorial choice task, CO = consumption. Column 5 reports the association between dependence severity (mainly indexed by self-reported drug use frequency, but see individual papers for details), and the proportion of drug versus alternative reward choice in a baseline condition, to test the economic decision model of dependence. Column 6 reports the association between dependence severity and the increase in drug choice (relative to intermixed no-stimulus control trials) produced by presenting drug associated cues in the Pavlovian to Instrumental Transfer (PIT) procedure, to test cue-reactivity theory. Column 7 reports the association between dependence severity and the decrease in drug choice (relative to baseline) produced by devaluing the drug outcome (through specific satiety, health warning or pharmacotherapy) in an extinction test, to quantify goal-directed control of drug choice, and test habit theory. Column 8 report the association between dependence severity and the reduction in drug choice (relative to baseline or intermixed control trials) produced by imposing costs on the drug choice (extinction, delay and opportunity costs), to test compulsion theory. Column 9 reports the association between dependence severity and the increase in drug choice (relative to baseline or intermixed control trials) produced by experimental mood induction (sadness or stress), to test negative reinforcement theory. Test statistics: F = general linear model; r = correlation coefficient; b = beta from multiple regression; t = t-test comparing high/low dependence subgroups; x^2^= Wald test; p = p value. The ns symbol = the association was reported as non-significant but the exact values were not published. The – symbol = the association was not tested.

1	2	3	4	5	6	7	8	9
Paper	Sample	Sample size	Choice task	Association between dependence severity & preferential choice of drug vs. alternative reward	Association between dependence severity & drug cue induced increase in drug choice	Association between dependence severity & drug devaluation induced decrease in drug choice	Association between dependence severity & cost induced decrease in drug choice	Association between dependence severity & mood induced increase in drug choice
[14].	Student drinkers	128	TE	**r = .38, p<.001**	F = .01, p = .93	–	–	–
[61] E1	Student smokers	24	TE	**r = .54, p = 0.01**	r = −.09, p = .66	–	–	–
[61] E2	Community smokers	128	TE	**r = .26, p = .004**	r = .01, p = .89	–	–	–
[62]	Student smokers	44	TE	**r = .52, p = .001**	ns	–	ns	–
[46]	Student smokers	91	TE	**r = .54, p<.001**	r=-.02, p = .91	**r=-.57, p<.001**	–	–
[63]	Student drinkers	62	TE	**r = .32, p<.05**	r=-.14, p>.05	r=-.21, p>.05	–	–
[64] E2	Student smokers	92	TE	**r = .54, p<.001**	r = .04, p = .68	r = .02, p = .87	–	–
[64] E1	Student smokers	64	TE	**r = .51, p<.001**	–	r = .08, p = .51	–	–
[65]	Student drinker	127	TE	**r = 2.8, p = .002**	–	–	F = 1.72, p = .19	–
[66]	Cocaine dependent	51	PC	**r = .38, p<.01**	–	–	ns	–
[33]	Student smokers	86	CO	**b = 1.59, p = .02**	–	–	**b = −42.92, p<.001**	–
[67] E1a	Student drinkers	127	PC	**r = .32, p<.001**	–	–	–	**x^2^ = 5.99, p = .01**
[67] E1b	Student drinkers	127	PC	**r = .23, p = .01**	–	–	–	**x^2^ = 13.78, p<.001**
[67] E2	Hazardous community drinkers	60	PC	**r = .29, p = .03**	–	–	–	**x^2^ = 9.76, p = .002**
[68]	Student drinkers	192	PC	**r = .43, p<.001**	–	–	–	**r = .16, p = .03**
[69]	Hazardous community drinkers	48	PC	**r = .42, p = .003**	–	–	–	r=-.03, p = .84
[56]	Student drinkers	128	TE	**r = .24, p = .007**	–	–	–	r=-.00, p = .97
[70]	Student drinkers	128	PC	**r = .31, p = .001**	–	–	–	r = .06, p = .54
[71]	Student smokers	100	TE	**r = .51, p<.001**	–	–	–	–
[7] E1	Treatment-seeking smokers	33	PC	**r = .45, p = .009**	–	–	–	–
[7] E2	Treatment-seeking drinkers	48	PC	**r = .59, p<.001**	–	–	–	–
[72]	Treatment-seeking smokers	207	PC	**r = .28, p<.01**	–	–	–	–
[73]	Community smokers	40	TE	**t = 4.12, p<.001**	–	–	–	–
[74]	Cocaine dependent	20	PC	**r = .62, p<.01**	–	–	–	–
[75]	Cocaine dependent	42	PC	**F = 15.3, p<.001**	–	–	–	–
[25]	Cocaine dependent	71	PC	**r = .31, p<.01**	–	–	–	–
[76]	Community smokers	26	CO	**x^2^ = 6.98, p<.01**	–	–	–	–

**Table 2:** List of studies that used concurrent choice tasks to measure the preference between a drug versus an alternative reward in a drug user sample, and tested whether psychiatric symptom severity is associated with drug choice in a baseline condition (column 6), and/or with the increase in drug choice produced by negative mood induction (column 7). Column 4 reports the psychiatric symptom tested in the association, D = Depression and A = anxiety. Note that some studies tested the association with both depression and anxiety in the same sample. For other notations see the caption for Table 1.

1	2	3	4	5	6	7
Paper	Sample	Sample size	Psychiatric symptom	Choice task	Association between psychiatric symptom severity & preferential choice of drug vs. alternative reward	Association between psychiatric symptom severity & mood induced increase in drug choice
[77]	Community smokers	27	D	PC	r = .15, p = .45	**F = 5.33, p = .03**
[70]	Student drinkers	128	D	PC	r = .06, p = .54	**r = .25, p = .007**
[67] E2	Hazardous community drinkers	60	D	PC	**r = .35, p = .007**	x^2^ = 3.48, p = .06
[67] E2	Hazardous community drinkers	60	A	PC	**r = .46, p<.001**	**X^2^ = 9.12, p = .003**
[56]	Student drinkers	128	D	TE	**r = .19, p = .04**	F = .55, p = .58
[7] E1	Treatment-seeking smokers	33	D	PC	**r = .41, p = .02**	–
[7] E2	Treatment-seeking drinkers	48	D	PC	**r = .39, p = .007**	–
[7] E2	Treatment-seeking drinkers	48	A	PC	**r = 57, p<.001**	–
[72]	Treatment-seeking smokers	207	D	PC	r=-.05, p>.05	–
[72]	Treatment-seeking smokers	207	A	PC	r = .02, p>.05	–
[69]	Hazardous community drinkers	48	D	PC	**r = .31, p = .03**	r = .03, p = .85
[68]	Student drinkers	192	D	PC	r = .14, p = .06	r = .00, p = .99
[68]	Student drinkers	192	A	PC	r = .05, p = .52,	r = .03, p = .69
[81] E1	Student smokers	42	A	PC	**r = .53, p<.001**	r=-.10, p = 53
[67] E1a	Student drinkers	127	D	PC	r = .07, p = .43	x^2^ = 1.01, p = .31
[67] E1a	Student drinkers	127	A	PC	r = .00, p = .96	x^2^ = 2.12, p = .15
[67] E1b	Student drinkers	127	D	PC	r=-.11, p = .21	x^2^ = .01, p = .91
[67] E1b	Student drinkers	127	A	PC	r = .01, p = .92	x^2^ = 1.01, p = .32

**Table 3:** List of studies that used concurrent choice tasks to measure the preference between a drug versus an alternative reward in a drug user sample, and tested whether self-reported drug use to cope with negative affect is associated with drug choice in a baseline condition (column 5), and/or with the increase in drug choice produced by negative mood induction relative to baseline or intermixed control trials (column 6), to test negative reinforcement theory. For notations see the caption for Table 1.

1	2	3	4	5	6
Paper	Sample	Sample size	Choice task	Association between drug use to cope with negative affect & preferential choice of drug vs. alternative reward	Association between drug use to cope with negative affect & mood induced increase in drug choice
[56]	Student drinkers	128	TE	**r = .22, p = .01**	**F = 7.33, p = .008**
[70]	Student drinkers	128	PC	**r = .31, p = .001**	**r = .25, p = .007**
[68]	Student drinkers	192	PC	**r = 0.37, p<.001**	**r = 0.19, p = .007**
[84]	Opiate dependent	46	PC	r = .20, p = .18	**r = .29, p<.05**
[67] E1a	Student drinkers	127	PC	**r = .27, p = .003**	**x^2^ = 9.37, p = .002**
[67] E1b	Student drinkers	127	PC	**r = .22, p = .01**	**x^2^ = 10.93, p = .001**
[67] E2	Hazardous community drinkers	60	PC	**r = .45, p<.0001**	**x^2^ = 8.84, p = .003**
[81] E1	Student smokers	42	PC	**r = .57, p<.001**	r=-.25, p = .11
[81] E2	Student smokers	55	PC	p = .09	**F = 6.54, p = .01**
[81] E3	Community smokers	218	PC	**b = .23, p = .001**	–
[69]	Hazardous community drinkers	48	PC	**r = .46, p = .001**	r = .13, p = .37
[72]	Treatment-seeking smokers	207	PC	**r = .22, p<.01**	–
[7] E1	Treatment-seeking smokers	33	PC	**r = .41, p = .02**	–
[7] E2	Treatment-seeking drinkers	48	PC	**r = .49, p<.001**	–

Table 1 lists 27 studies which tested whether dependence severity within a drug user group was associated with preferential choice of a drug versus alternative reward in a concurrent choice task, to test the economic decision model of dependence. As shown in column 5, in all 27 studies, dependence severity within the user group was significantly associated with greater drug choice, suggesting dependence is underpinned by greater relative value of the drug versus alternative reward. Examination of column 2 indicates that this association has been found with different samples, including student, community and treatment-seeking users of different drug classes including alcohol, tobacco and cocaine. The association has also been found when using token economy and pictorial concurrent choice tasks suggesting both versions are sensitive to individual differences in drug value (column 4). The robustness of the association between dependence severity and drug choice across multiple designs and samples suggests that greater relative drug value may underpin dependence universally, consistent the economic demand task, and with the economic decision model.

Table 1 columns 6–9 list studies which tested the impact of an experimental manipulation (drug cues, outcome devaluation, costs, and negative mood induction) on drug choice, to determine if dependence severity was associated with sensitivity to these manipulations (for a meta-analysis of experimental manipulations impacting drug demand metrics see [35]). The studies in column 6 tested the prediction of cue-reactivity theory, that dependence would be associated with greater sensitivity to cue-induced increases in drug choice. The studies in column 6 demonstrated that presentation of drug related pictorial cues can increase drug choice compared to intermixed no-cue control trials in the Pavlovian to Instrumental Transfer (PIT) procedure [36]. The PIT test phase is designed to isolate the specific mechanism by which drug stimuli prime drug choice. In the test trials, the drug stimulus is presented for the first time while the two responses are available, and the test is conducted in extinction, where responses no longer earn their rewards. This design ensures that drug stimuli can only prime drug choice via an expectation (or inference) that drug response is more viable in the presence of the drug stimulus. The drug stimulus cannot prime drug choice through an S-R association because the stimulus has never previously been paired with the response, and testing in extinction ensures that this pairing is not reinforced during the test phase [37]. Consistent with this expectancy account, the drug PIT effect increases with participants’ beliefs that drug cues signal a greater probability of the drug choice being rewarded, and can be abolished by instructions which contradict this belief [14,38,39]. These findings suggests that drug cues modulate the expected (verbalizable) probability that the drug choice will produce the drug, which increases the likelihood of selecting the drug choice, consistent with prospect theory and other decision models that envisage a role for expected response-reward probabilities in action selection [37,40]. Crucially for cue-reactivity theory, however, as summarised in Table 1 column 6, dependence severity was not associated with a greater drug PIT effect in all seven studies. Each of these studies demonstrated a significant drug PIT effect (i.e. the drug cue increased drug choice relative to no cue trials), but the magnitude of the PIT effect was not correlated with dependence severity in any of these studies. A related set of findings have been reported with the cue-induced craving model, where drug cues have been shown to increase self-reported (verbalised) desire for the drug to the extent that those cues signal the probability that drug choice will produce the drug [41]. Again, however, cue induced increases in subjective craving are not reliably associated with dependence severity [42,43]. The conclusion, therefore, is that drug cues increase drug choice by raising the expected probability of receiving the drug (consistent with the unified decision making model), but sensitivity to this effect does not appear to play a role in vulnerability to dependence, an observation that contradicts cue-reactivity theory of dependence. This conclusion is supported by animal studies [44].

Table 1 column 7 lists studies which have measured the impact of drug devaluation on drug choice, to test habit theory of addiction. In these designs, baseline drug choice was first measured using a concurrent choice token economy procedure. Then, the drug or alternative reward was devalued by specific satiety, taste adulteration or health warnings. Finally, choice was measured again, but in extinction where the responses no longer earned their rewards such that choice could not be modified by direct experience of the rewards during the test. In the studies listed, drug devaluation reduced drug choice in the extinction test, demonstrating that drug choice is goal-directed in being controlled by an expectation of the current value of the drug plus knowledge of the response-outcome contingencies [45]. Stated another way, in the absence of feedback from outcomes earned in the extinction test, there is nothing to guide choice other than expectations about the current value of the outcomes previously earned by each response. Habit theory predicts that dependence should be associated with *insensitivity* to drug devaluation, because drug choice is controlled by an autonomous S-R association established in initial training, rather than by an expectation of current value of the drug [16], such that devaluation should produce no change in drug choice in the extinction test. However, in all four studies that tested this prediction, dependence severity was not associated with insensitivity to the devaluation effect, contradicting habit theory. In the one study which did show a significant association [46], the devaluation treatment was 1 mg of intra nasal nicotine replacement therapy (NRT), which produced a significant increase in goal-directed tobacco choice in more dependent smokers (i.e. a priming effect), and produced a significant decrease in goal-directed tobacco choice in less dependent smokers (i.e. a satiety effect). This finding contradicts habit theory which predicts *insensitivity* to devaluation treatment in more dependent individuals. On the basis of these outcome devaluation studies, human dependence severity does not appear to be associated with a propensity to habit learning (insensitivity to drug devaluation). Other studies not included in Table 1, which tested whether drug users versus non-user control participants differed in their sensitivity to devaluation manipulations have similarly failed to find any consistent group difference, again providing little or no evidence for habit theory [22,[47], [48], [49]].

Table 1 column 8 lists studies that measured the impact of imposing costs on drug choice, to test compulsion theory. In these designs, baseline drug choice was measured, and then a cost was imposed on the drug choice in test trials, either by increasing the number of responses needed to obtain the drug reward (extinction), adding a delay between the response and receipt of the drug reward, or by increasing the value of the alternative natural reward that would be missed as a consequence of choosing the drug (opportunity costs). Although these types of costs are qualitatively different (and are grouped together largely for convenience), in each study, the imposition of costs significantly reduced drug choice, indicating that drug choice is an economic decision based on a rational evaluation of the payoff [50]. Most importantly, dependence severity was not associated with impaired sensitivity to the impact of costs on drug choice in three out of four of the studies, providing no evidence for compulsion theory. In the one study which found a significant association [33], increasing the value of the money alternative decreased tobacco choice more steeply as a function of tobacco dependence severity (converse to the prediction of compulsion theory), due to more dependent smokers choosing tobacco more frequently when the value of money was low. Thus, these concurrent choice studies contradict the prediction of compulsion theory that dependence is driven by a maladaptive S-R association that renders drug-seeking invulnerable to immediate punishment [17] (for a more extensive critique of compulsion theory see [22,51]). It is interesting to note that other studies that tested the impact of imagined next day responsibilities on measures of drug demand (willingness to pay for the drug), found evidence that dependence may be linked to insensitivity to these future costs [[52], [53], [54], [55]]. Thus, dependence may be linked to an inability to incorporate future costs into decision making consistent with delay discounting models of addiction [21], but there is little evidence that dependence is linked to insensitivity to immediate costs, an observation that is inconsistent with compulsion theory [17]. Future work might test this prediction by contrasting the effect of immediate versus imagined future costs on concurrent drug choice to determine whether dependence is uniquely associated with reduced sensitivity to imagined future costs.

Finally, Table 1 column 9 lists studies which measured the impact of negative mood induction (sadness or stress) on drug choice to test negative reinforcement theory. In these studies, drug choice was measured in baseline trials, and then negative affect was induced across a test block, or acutely primed in intermixed test trials. In each study, mood induction increased drug choice indicating that mood induction preferentially raised the value of the drug versus alternative reward. Importantly, mood induction can prime drug choice in the extinction test, indicating that negative mood raises expected drug value driving increased goal-directed drug choice [56,57]. Furthermore, mood induction can fully countermand the effects of drug devaluation (by specific satiety) on goal-directed drug choice [57] suggesting the two motivational states have opposite effects on expected drug value, and that mood induction is more powerful than satiety – a primary motivational state. The most important observation, however, is that dependence severity was associated with greater sensitivity to mood induced increases in drug choice in four out of the eight studies that investigated this, as summarised in Table 1 Column 9. Although this association was only demonstrated in half of the studies that tested it, the positive studies provide initial support for the claim of negative reinforcement theory that sensitivity to negative mood induced drug choice plays a role in vulnerability to dependence [[58], [59], [60]]. To explore this further, the next two tables examine whether sensitivity to mood induced drug choice is more closely associated with psychiatric symptoms and self-reported drug use to cope with negative affect.

Negative reinforcement theory predicts that substance users who have psychiatric symptoms should ascribe greater relative value to the drug (i.e. show greater baseline drug choice) and should be more sensitive to acute negative mood induced increases in drug choice (because these individuals have learned that the drug mitigates psychiatric symptoms and acute negative affect) [58]. Table 2 Column 6 summarises studies which have tested whether psychiatric symptom severity (depression and anxiety) within a drug user group is associated with preferential drug choice in a concurrent choice task. As shown, psychiatric symptoms were associated with baseline drug choice in eight out of 18 tests. The association appears to be weak in students (2/9 positive), but stronger in community/treatment-seeking samples (6/9 positive) especially if they are drinkers (5/5 positive) but not smokers (1/4 positive). It may be that psychiatric symptoms only promote drug choice beyond a certain level of symptom severity (i.e. the relationship is non-linear), or period of learning has passed (age dependency), or students may have protective factors. There may also be differences between drug classes. Isolating these potential moderators of the association will require further studies and meta-analysis to uncover.

Table 2 Column 7 summarises studies which have tested whether psychiatric symptoms are associated with greater sensitivity to negative mood induced increases in drug choice. This association was found in only three out of 13 tests, suggesting the associations may only be found under specific conditions – but the moderating variables are far from obvious. One possibility is that the association may be found when the negative state induced experimentally is relevant to the sample’s unique psychiatric symptom profile. In one study with adult daytime pub drinkers, a noise stress induced increase in alcohol choice was associated with depression marginally, but anxiety significantly, and this association survived when controlling for depression, suggesting a unique association between stress induction and anxiety [67]. Conversely, an association has been found between sadness induced drug choice and depression in smokers preselected with low versus high depression symptoms [77] and in students drinkers [70], suggesting there may a unique association between sadness induction and depression. There remains the question of why similar studies failed to find such associations. There may be other factors such as the questionnaires used to assess psychiatric symptoms (i.e. whether they are sensitive in student and clinical samples, or tap the correct construct), as well as possible randomness in sampling resulting in different levels of symptom severity between studies. Our working hypothesis is that future work might benefit from exploring the unique associations between specific psychiatric symptoms and specific experimental mood induction protocols [67,[78], [79], [80]].

Negative reinforcement theory predicts that substance users who report using substances to cope with negative affect should ascribe greater relative value to the drug (i.e. show greater baseline drug choice) and should be more sensitive to acute negative mood induced increases in drug choice (because these individuals have learned, and verbally report, that the drug mitigates negative affect) [22]. Although self-reported substance use to cope with negative affect is closely associated with psychiatric symptoms, coping motives are thought to proximally mediate drug consumption decisions [82,83]. If this is true, we would expect coping motives to be more reliably associated with drug choice, and negative mood induced drug choice, than psychiatric symptoms. Consistent with this view, Table 3 Column 5 shows that 12 out of 14 studies found that coping motives were associated with drug choice, and the two nonsignificant associations were marginal, giving a higher ratio of positive findings than psychiatric symptoms. Furthermore, as shown in Table 3 Column 6, coping motives were associated with negative mood induced drug choice in eight of the 10 studies, again demonstrating a more replicable association than was found with psychiatric symptoms. These associations were not only reliable, but also generalizable in being found across drug classes (alcohol, tobacco, opiates), with student, community and treatment-seeking samples, and with different choice protocols (token economy and pictorial choice). These findings strongly suggest that substance use coping motives are a risk factor for greater drug value, and greater sensitivity to mood induced increases in drug value.

A key study indicates that negative mood induced drug choice is goal-directed, i.e. driven by an increase in expected drug value [56]. This study found that negative mood statements versus randomly intermixed neutral statements primed alcohol over food choice when these statements were presented for the first time in an extinction test (i.e. where the responses no longer earned their rewards). This effect is most feasibly explained by suggesting that negative mood statements acutely retrieved an expectancy (or inference) that the alcohol reward currently has a higher value, which augmented goal-directed alcohol choice. The effect cannot be readily explained by S-R or other automatic priming theories because the negative mood statements had never been directly paired with alcohol response, and this pairing was not rewarded during the extinction test, so no S-R association could form (see also [57]). Crucially, the effect was more pronounced in individuals who reported alcohol use to cope with negative affect, indicating that negative affect raises expected drug value promoting goal-directed drug choice to a greater extent in these individuals. This is precisely the sort of risk pathway that would be anticipated by a unification of economic decision and negative reinforcement theories of addiction.

The human concurrent choice findings reviewed above provide insight into the type of unified economic decision theory that might explain vulnerability to addiction. Our observations were that drug choice could be modified by a variety of distinct decision variables, namely drug cues, drug devaluation, costs (response requirements, delay and opportunity costs), and negative mood induction (stress and sadness). Indeed, two of these decision variables – devaluation and negative mood induction – were shown to have opposing effects on drug choice when compounded, demonstrating a convergence of decision variables on behaviour [57]. More work is needed to test additive and subtractive effects of compounding decision variables on drug choice to establish the weight assigned to each decision variable [85]. Crucially, choice between the drug and alternative was demonstrated to be goal-directed, that is, controlled by an expectation of the drug, rather than determined by an S-R association or other automatic process. Drug cue priming of drug choice, devaluation induced decreases in drug choice, and negative mood induced increases in drug choice were all shown to be driven by changed expectations about the drug (in the extinction test of the outcome devaluation/revaluation procedure [86]). The implication is that drug choice is controlled by expectations about the drug’s value in the context of the individual’s current internal state, expectations about the probability of the response-reward contingency being in force given the external stimulus context, and expectations of the costs/risks arising from the drug choice. The convergence of these multiple distinct decision variables on drug expectancy may be the mechanism by which these factors are combined (commensurated) into a common neural currency to impact drug choice behaviour [87,88].

**Fig. 1:**
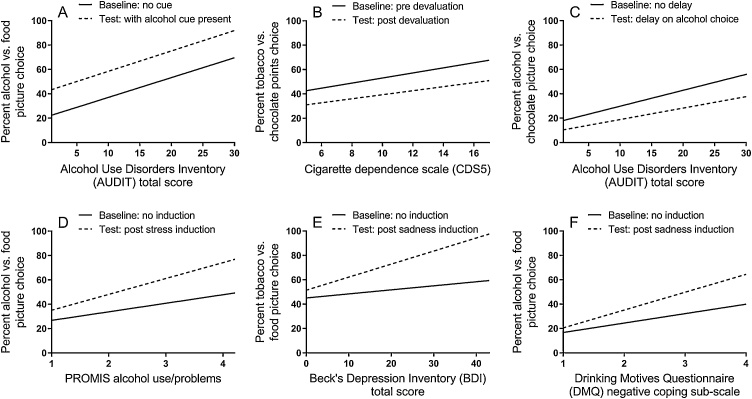
Figure 1 shows the regression slopes relating percent choice of the drug versus alternative reward as a function of three individual difference factors – dependence, depression and drug use coping motives – measured under baseline conditions (black line) and test conditions (dashed line) which tested the impact of different decision variables on drug choice (A-F). The associations illustrate the main conclusions drawn from the exhaustive review of concurrent choice association studies listed in Tables 1-3. Dependence severity was associated with preferential drug choice in baseline conditions (A-C), but not with greater sensitivity to a cue-induced increase in drug choice (A [14]) as indicated by the lack of interaction between slopes, contradicting cue-reactivity theories of addiction. Similarly, dependence severity was not associated with insensitivity to the decrease in drug choice produced by drug devaluation (B [64]) or by imposing costs on the drug choice (C [65]), contradicting habit and compulsion theories of addiction, respectively. By contrast, greater sensitivity to negative mood induced increases in drug choice was associated with dependence (D [67]) and depression (E [77]), but most reliably with self-reported drug use to cope with negative affect (F [70]). The implication is that vulnerability to dependence is conferred by greater relative value ascribed to drugs, and relative drug value is further augmented by negative affective states in those who report drug use coping motives. Dependence does not appear to be characterised by abnormal cue-reactivity, habit learning or compulsion..

Fig. 1 summarises our conclusions about individual differences in sensitivity to decision variables influencing drug choice drawn from Table 1, Table 2, Table 3. The first observation is that dependence severity was reliably associated with preferential drug choice in all 27 studies that investigated this. Fig. 1A provides one example of this association. Dependent individuals might choose the drug more frequently because they hyper-value the drug or hypo-value the alternative rewards, or both, but either way, they ascribe greater relative value to the drug. The addition of 85 studies showing that dependence is correlated with greater economic demand for drugs provides additional compelling evidence that dependence is driven by greater relative drug value.

The second observation is the absence of an association between dependence severity and drug cue priming of drug choice in all seven studies that investigated this. Fig. 1A provides one example finding [14]. The drug cue raised drug choice overall, i.e. a main effect between slopes. However, there was no interaction between dependence severity and the slopes, indicating that more dependent individuals are not more sensitive to drug cue priming of drug choice, contradicting cue-reactivity theories of dependence. The same was true regarding the effect of devaluation – dependence severity was not associated with insensitivity to devaluation in all four studies that tested this, contradicting habit theory. Similarly, dependence severity was not associated with insensitivity to direct costs imposed on the drug choice in all four studies that tested this, contradicting compulsion theory. These null associations can be seen in Fig. 1B [64] and C [65], where there were main effects between slopes, indicating that devaluation and imposition of costs reduced drug choice. However, there were no reliable interactions, indicating that dependence was not associated with differential sensitivity to devaluation and costs, contradicting habit and compulsion theories, respectively.

By contrast, mood induced increases in drug choice was more reliably associated with individual differences. Mood induced drug choice was associated with dependence severity in four of the seven studies that investigated this, with psychiatric symptoms in three out of 13 tests of this association, and with self-reported drug use to cope with negative affect in eight out of 10 studies. Examples of these associations are shown in Fig. 1D-F [67,70,77]), where there was an interaction between slopes indicating differential sensitivity to negative mood induced drug choice across the individual difference factor. Mood induced drug choice was more replicably associated with coping motives than with dependence severity or psychiatric symptoms, suggesting that coping motives is the most proximal determinant of this sensitivity. A related conclusion can be drawn from mediation analyses of survey data (Reviewed in [22] and [83]). These mediation studies show that multiple adverse states (including, stress, depression, anxiety, financial strain, antisocial behavior, bullying, intimate partner violence, intimate partner sexual coercion, childhood physical/sexual/emotional abuse, childhood trauma and adult trauma) are all associated with dependence severity, and this relationship is mediated by self-reported use of drugs to cope with negative affect. The implication is that coping motives underpin dependence in those who have suffered adversity. Our overall conclusion is that vulnerability to dependence is driven by individuals ascribing greater expected relative value to the drug driving greater goal-directed drug choice, and there is an additional risk pathway for individuals who develop drug use coping motives (as a result of adversity), which confers sensitivity to negative affective states further raising expected relative drug value, promoting even greater goal-directed drug choice. By contrast, vulnerability to dependence is not driven by greater sensitivity to cue-induced drug choice (cue-reactivity), insensitivity to devaluation (habit learning) or insensitivity to costs imposed on drug choice (compulsion).

## 3 Changes in the relative value of drugs versus alternative rewards may underpin recovery from addiction

Many people recover from addiction, often without any formal treatment [89] and there are a number of psychological and pharmacological treatments for addiction that have demonstrable efficacy [90,91]. Any coherent theoretical account of addiction must be able to explain how people with addiction are able to recover, and how treatments exert their beneficial effects. As recently outlined elsewhere [6], behavioural economic accounts that emphasise the centrality of the relative valuations of drugs versus competing non-drug reinforcers offer a parsimonious account of recovery and the mechanisms of action of treatment. These accounts are focussed on non-drug reinforcers that act as substitutes rather than complements for drug reinforcement [11,92]. The importance of non-drug substitute reinforcers in behavioural economic demand and choice has been largely overlooked, although it has been recognised in recent human [93] and animal [94] addiction research. In this section we provide a brief overview of the literature on behavioural economic factors in recovery from addiction and in the mechanisms of action of treatments.

In humans, recovery from addiction occurs when the availability of drug-free rewarding activities increases [6,[95], [96], [97], [98]] and/or when the detrimental effects of drug use on health and interpersonal relationships become more salient [99]. Comparable findings have been reported in animal models of drug use and its cessation [34,100]. To our knowledge there are no prospective studies that tracked changes in behavioural economic demand for the drug or changes in the valuation of drug versus non-drug rewards during unassisted recovery from addiction. However in a recent cross-sectional study we demonstrated that individual differences in “meaning in life” (which might loosely correspond to valuations attached to non-drug activities) were negatively associated with scores on the Alcohol Use Disorders Identification Test, and this association was partially mediated by indices of behavioural economic demand for alcohol derived from the Alcohol Purchase Task [101]. Longitudinal studies are required to investigate if unassisted resolution of drinking problems and “maturing out” of alcohol use disorders and other addictions are accompanied by and mediated by changes in economic demand for the drug and the relative valuation of the drug versus competing reinforcers.

Regarding the mechanisms of action of established treatments for addiction, some studies have implicated changes in behavioural economic demand as important mediators of the effects of a variety of psychological interventions on drug use outcomes. For example, brief motivational interventions and pharmacotherapy prompted changes in behavioural economic demand for the drug, and these changes in turn predicted the likelihood of sustained changes in drug use after receiving the treatment [102,103]. Another study found that shifts in the proportionate reinforcement derived from drug use versus drug-free activities accounted for the effects of a motivational intervention on alcohol dependence [104]. In a recent paper [6] we argued that changes in the relative valuation of drugs versus drug-free alternatives (behavioural economic substitutes) may mediate the beneficial effects of diverse interventions on drug use. For example, mindfulness techniques that train people to ‘savour’ experiences associated with substance-free activities in their lives may increase the value attributed to substance-free alternatives [105]. Another example relates to the changes in social networks that are associated with involvement in Alcoholics’ Anonymous, in which people in recovery distance themselves from heavy drinkers in their social networks and replace them with other people who are also in recovery, which should in principle shift the relative valuation of alcohol versus alcohol-free alternative activities [106]. To give another example, cognitive behavior therapy may partially exert its beneficial effects because it encourages people with addiction to think about how their drinking is hurting family members (thereby devaluing alcohol), and counterconditioning exercises may increase the valuation of substance-free alternatives [107].

The effectiveness of contingency management and related interventions provides more direct evidence that changing the valuation of drugs versus drug-free alternatives plays an important role in the successful treatment of addiction. Contingency management approaches offer financial incentives to people with addiction if they can confirm their abstinence from drugs, which should in principle devalue the drug whilst increasing the value of drug-free activities that the financial incentives can be used to obtain. These approaches lead to reductions in drug use [108] that may be mediated by the extent to which they rebalance the relative value of drug use versus drug-free alternative behaviours [109,110]. More recently studied interventions include behavioural activation and substance-free activity sessions that aim to increase the availability and value of drug-free activities, thereby indirectly reducing the *relative* value of drugs. These interventions lead to reductions in drug use that may be mediated by increases in engagement in drug-free activities [104,111,112]. A speculative interpretation of these findings is that shifts in the relative valuation of drugs versus competing reinforcers may underlie these effects, an interpretation that could be evaluated in future studies of the mediators of behavior change after these types of treatments [113].

In this section we have outlined how changes in valuations of drugs and drug-free alternatives may at least partially account for how diverse treatments for addiction exert their therapeutic effects, and how people recover from addiction. We acknowledge that this is speculative, and further work with validated measures of valuation (such as the concurrent choice and alcohol purchase tasks) is needed to confirm and build on these findings. We also acknowledge that our focus on the centrality of valuation processes may appear to overlook other aspects of addiction treatment and recovery such as self-efficacy and the acquisition of coping skills that may play an important role in the valuation processes. These important constructs may be reconciled with a valuation-based account if one considers the internal mechanics of value-based decision-making. We describe computational work on value-based decision-making in the next section.

## 4 The internal mechanisms that underpin value-based decisions, and distortion of those mechanisms in addiction

Concurrent choice tasks in which participants choose between a drug versus alternative reward have contributed important information about the role of decision making in addiction [7,114]. However, this task leaves some uncertainties regarding the internal processes that precede and determine overt choice. Computational models of value-based decision making help characterise the accumulation of evidence prior to a choice being made – and how this process might be distorted in addiction. More specifically, a focus on the internal machinery of value-based decision-making may enable a unified decision theory that can account for how drug related cues, devaluation, imposition of costs and negative mood induction might influence overt behavior through a common mechanism.

Experimental procedures for assessing value-based decision-making (VBDM) are similar to those used in the concurrent choice tasks described above: Participants must choose between two pictures that depict valued outcomes. However, there are a number of important differences. Firstly, in VBDM procedures participants initially view a battery of images and they are asked to report the degree to which they would most like to consume or experience each one – this is important for rank ordering the images in each category from least to most valued [115]. If VBDM procedures were to be applied to the study of addiction, this initial subjective rating phase would be done separately for drug images (e.g. pictures of different alcoholic drinks, or scenarios that involve drinking alcohol) and non-drug images (e.g. images that depict valued objects or activities that do not involve alcohol, such as spending time with one’s children, engaging in hobbies or sport, etc.). After participants provide these ratings, they complete a concurrent choice task in which either two drug-related images or two non-drug images are presented side-by-side, and participants must select their preferred option as quickly as possible. The initial picture ratings are important because these are used to determine pairs of images that are presented on each trial: some trials might be relatively ‘easy’ for participants (for example, if they have to choose between a highly preferred and a least preferred image), whereas other trials might be more difficult for participants (for example if they have to choose between two images that were both rated similarly during the initial rating task). It is also important to note that, unlike the concurrent choice task described above, participants are never asked to choose between drug and non-drug options: instead, VBDM parameters must be extracted separately from drug picture and non-drug picture blocks.

Participants’ reaction time and ‘error’ data (with errors inferred if participants’ selections during the choice task are inconsistent with their stated preferences during the initial rating task) are then fitted using drift diffusion models [116,117]. These models operate on the basic assumption that, when faced with a particular choice set (e.g. between two images that depict drug outcomes), internal evidence for each possible outcome accumulates over time until the accumulated evidence for one crosses a response or decision boundary. Evidence (or value) accumulation is noisy, which determines the frequency of errors that participants make, along with the characteristic distribution of reaction times. Fitting of drift diffusion models to participants’ reaction time and error data enables the recovery of distinct parameters that underlie value-based choice. These parameters include the rate of evidence (or value) accumulation for the category of stimuli that are presented during the task (for example, pictures depicting the participants’ drug of choice, or pictures that depict drug-free rewards) and the decision-maker’s response threshold when they are making choices between images that belong to that particular category.

According to a recent conceptual model [6,118] these VBDM models can be applied to account for addiction in general, and for performance on classic concurrent choice tasks [119] as follows. Firstly, consider how people would be expected to perform on a VBDM task with alcohol-related images. On this task, the person with alcohol dependence should have a higher rate of evidence accumulation and a lower response threshold, compared to a person who drinks infrequently and is not dependent on it. By contrast, if those same two individuals were to perform a VBDM task with images that depict non-drug rewarding activities (for example, going for a walk in the countryside), we would expect to see the opposite pattern: a higher rate of evidence accumulation and a lower response threshold in the person who drinks alcohol infrequently and is not dependent, compared to the person who is dependent on alcohol. In this way, the VBDM task has the potential to isolate drug hypervaluation and alternative reward hypovaluation, unlike the concurrent choice task which conflates these two processes. However, the VBDM task lacks a direct comparison of drug versus alternative rewards, which may be important to isolate the core dysfunction in addiction. Perhaps then, the best evidence on the role of VBDM in addiction would come from a convergence of both types of task.

Application of VBDM as a tool to explain vulnerability to addiction and recovery from it can be used to identify novel hypotheses regarding the mechanisms through which some people are particularly vulnerable to addiction, and how they eventually recover. Regarding vulnerability to addiction, prospective studies could investigate if future dependence severity is best predicted by elevated evidence accumulation for the drug, suppressed evidence accumulation for non-drug alternatives, a low response threshold when evaluating drug options, an elevated response threshold when evaluating non-drug options, or if each of these parameters is equally predictive of dependence in the future. We also noted earlier that coping motives are associated with increased sensitivity to negative mood induction raising the expected relative value of the drug, augmenting goal-directed drug choice. It would be interesting to test whether this mood induction effect was due to elevated evidence accumulation for the drug, suppressed evidence accumulation for non-drug alternatives, a low response threshold when evaluating drug options, and / or an elevated response threshold when evaluating non-drug options. Similarly, future work could investigate whether the impact of other experimental manipulations, including the presentation of drug cues in the PIT task, drug devaluation, and the imposition of costs on the drug choice, can be explained by changes in evidence accumulation rates or response thresholds for the drug versus alternative reward.

Regarding recovery from addiction, in our recent paper we outlined the changes to VBDM parameters that we would expect to see as people receive treatment for addiction and recovery [6]. For example, interventions such as behavioural activation [111] that aim to directly increase the value of drug-free activities should exert their beneficial effects on drug use because they amplify the rate of evidence accumulation when participants are choosing between two valued drug-free alternatives, but this intervention should have no effect on the rate of evidence accumulation when participants are choosing between two drug alternatives. By contrast, a pharmacological intervention such as nicotine replacement therapy [120] would be expected to selectively suppress the rate of evidence accumulation when participants are choosing between two drug alternatives. Finally, elements of mindfulness interventions that focus on “acceptance of uncomfortable states or challenging situations without reacting automatically” [121] might exert their beneficial effects by raising the person’s response threshold when they are faced with an opportunity to use the drug, which might be sufficient for them to resist drug use on that occasion. Such application of VBDM tasks to understand addiction, its treatment, and recovery is speculative and awaits empirical testing.

## 5 Summary and conclusion

We started with the premise of behavioural economic theory of addiction, that greater expected value of drug relative to alternative non-drug rewards is the core mechanism underpinning vulnerability to and recovery from addiction. We evaluated this claim by reviewing human concurrent choice studies with drug users, which showed that dependence severity was reliably associated with overall drug preference, and self-reported drug use to cope with negative affect was reliably associated with greater sensitivity to mood induced increases in drug choice, suggesting these two risk pathways underpin addiction. By contrast, addiction severity was not consistently associated with sensitivity to the effect of drug cues, drug devaluation or punishment on drug choice, an observation which contradicts cue-reactivity, habit and compulsion theories of addiction. We then described evidence that therapeutic interventions and recovery from addiction might be attributed to changes in the expected relative value of drug versus alternative rewards. In the final section, we outlined a speculative computational model and novel experimental method that could be used to probe accumulation of evidence about drugs and alternative rewards that could drive action selection. Combining the standard concurrent choice paradigm and these new computational approaches will be fruitful areas for future research, improving the understanding and treatment of addiction.

## Funding source

The research was supported by an 10.13039/501100012450Alcohol Change UK grant (RS17/03) to Lee Hogarth, and a Medical Research Council (UK, MRC) Confidence in Global Mental Health pump priming award (MC_PC_MR/R019991/1) to Hogarth. Funders had no role in the study design, collection, analysis or interpretation of the data, writing the manuscript, or the decision to submit the paper for publication.

## Declaration of Competing Interest

None.
